# Expression of Potato *StDRO1* in Arabidopsis Alters Root Architecture and Drought Tolerance

**DOI:** 10.3389/fpls.2022.836063

**Published:** 2022-05-19

**Authors:** Chao Sun, Wenjun Liang, Kan Yan, Derong Xu, Tianyuan Qin, Sajid Fiaz, Philip Kear, Zhenzhen Bi, Yuhui Liu, Zhen Liu, Junlian Zhang, Jiangping Bai

**Affiliations:** ^1^Gansu Provincial Key Laboratory of Arid Land Crop Science, College of Agronomy, Gansu Agricultural University, Lanzhou, China; ^2^School of Biological and Pharmaceutical Engineering, Lanzhou Jiaotong University, Lanzhou, China; ^3^Department of Plant Breeding and Genetics, The University of Haripur, Haripur, Pakistan; ^4^International Potato Center (CIP), CIP China Center for Asia Pacific (CCCAP), Beijing, China

**Keywords:** molecular breeding, root system, branch angle, abiotic stress, food security

## Abstract

Potato (*Solanum tuberosum* L) is the third important crop for providing calories to a large human population, and is considered sensitive to moderately sensitive to drought stress conditions. The development of drought-tolerant, elite varieties of potato is a challenging task, which can be achieved through molecular breeding. Recently, the *DEEPER ROOTING 1* (*DRO1*) gene has been identified in rice, which influences plant root system and regulates grain yield under drought stress conditions. The potato StDRO1 protein is mainly localized in the plasma membrane of tobacco leaf cells, and overexpression analysis of *StDRO1* in Arabidopsis resulted in an increased lateral root number, but decreased lateral root angle, lateral branch angle, and silique angle. Additionally, the drought treatment analysis indicated that *StDRO1* regulated drought tolerance and rescued the defective root architecture and drought-tolerant phenotypes of *Atdro1*, an Arabidopsis *AtDRO1* null mutant. Furthermore, *StDRO1* expression was significantly higher in the drought-tolerant potato cultivar “Unica” compared to the drought-sensitive cultivar “Atlantic.” The transcriptional response of *StDRO1* under drought stress occurred significantly earlier in Unica than in Atlantic. Collectively, the outcome of the present investigation elucidated the role of DRO1 function in the alternation of root architecture, which potentially acts as a key gene in the development of a drought stress-tolerant cultivar. Furthermore, these findings will provide the theoretical basis for molecular breeding of drought-tolerant potato cultivars for the farming community.

## Introduction

Potato (*Solanum tuberosum*) is indispensable for food security around the globe and the fourth largest food crop in China (Cao et al., 2020). The world potato catalog contains information on approximately 4,500 cultivable varieties from around the globe.^1^ These potato cultivars vary by various morphological, physiological, biochemical, and pathological attributes under ever-changing environmental conditions (Pieczynski et al., 2018). Several classical and molecular studies have been undertaken to understand the genomic regions controlling traits with agricultural and economic importance using diploid and tetraploid potato plants. Kondrák et al. (2012) developed a transgenic White Lady potato cultivar, which expressed the *trehalose-6-phosphate synthase* gene exhibiting drought tolerance. Similarly, Zaki and Radwan (2022) investigated a set of 21 commercial potato cultivars representing genetic diversity in the Middle East and screened drought tolerance based on morpho-physiological traits and tuber production under *in vitro* and field trails. The results displayed the upregulation of *DRO, ERECTA, ERF, DREB*, and *StMYB* genes in drought-tolerant cultivars, indicating the possible role of these genes in future molecular breeding programs. Recently, the availability of genome sequence data for most crops, e.g., Arabidopsis (Weigel and Mott, 2009), rice (3, 000 rice genomes project, 2014), wheat (Walkowiak et al., 2020), soybean (Xie et al., 2019), maize (Vicki and Volker, 2002), and potato (Leisner et al., 2018; Hoopes et al., 2022) has enabled to understand and improve both quantitative and qualitative traits, especially genes governing abiotic stress tolerance.

Drought is considered the major abiotic stress for crop plants (Sun et al., 2021). The availability of irrigation water will continue to decrease across the globe owing to a surge in human population from 7 to 9 billion by 2050 (Edmeades, 2013). Therefore, it is imperative to use agricultural mechanization and cultivation water-saving techniques, as well as to develop high-yield and high-quality varieties with better resistance to biotic and abiotic stresses to improve agricultural production (Weber et al., 2014; Brito et al., 2016). Potato, being a shallow root crop, is relatively more sensitive to drought stress than other staple crops (Deblonde and Ledent, 2001; Schafleitner et al., 2007). Long-term or seasonal drought seriously affects the yield and commercial quality of potato (Walworth and Carling, 2002). Moreover, it is notable that some major potato production areas are located in arid and semiarid regions (Porter et al., 1999; Fabeiro et al., 2001). Accelerating global climate change and associated drought is a threat to potato production (Kumar et al., 2007; Li et al., 2019). Roots are integral in performing a variety of functions, e.g., nutrients and water uptake, serving as a storage organ and helping the plant to anchor in the soil (Smith and De Smet, 2012). The variable interactions of plant roots with the environment depends on root components and root architecture (Lynch and Brown, 2012).

Root architecture defines the spatial configuration of roots and helps the plant to respond to ever-changing environmental conditions. Understating root architecture is important for agricultural productivity because mostly soils have an uneven distribution of resources (Zhao et al., 2018). The spatial distribution of roots allow the plant to exploit available soil resources efficiently. Plant roots function in the absorption and transport of water and nutrients, and root architecture is known to strongly contribute for plant’s ability to tolerate abiotic stresses, especially drought condition (Manschadi et al., 2006; Mansoor-khani et al., 2014; Bartlett et al., 2022; Ranjan et al., 2022; Rasool et al., 2022). Several studies have shown that drought (or the lack of irrigation in the topsoil) can promote the formation of deeper roots to allow crops to access water and nutrients from the deeper soil (Shahnazari et al., 2007; Chimungu et al., 2014). In recent years, extensive efforts have been observed to harness deep rooting architecture as a screening and evaluation index for drought-tolerance breeding in some cereal crops (Wasson et al., 2012; Lynch, 2013; Liao et al., 2022). Genetic information focused on root architecture, and its role to counter abiotic stresses especially drought in tuber crops is less available (Villordon et al., 2014a).

In rice, a major quantitative trait locus, *OsDRO1* (*DEEPER ROOTING 1*), was functionally characterized by map-based cloning of two varieties with apparent differences in their root architecture. The DRO1 protein was shown to regulate both root angle and drought tolerance (Uga et al., 2012, 2013; Arai-Sanoh et al., 2014). Subsequently, *DRO1* orthologs in Arabidopsis (*Arabidopsis thaliana*), plum (*Prunus domestica*), and wheat (*Triticum aestivum*) were also found to function in regulating root architecture; however, it is notable that the specific root traits regulated by this gene were distinct in these plants (Hollender and Dardick, 2015; Ge and Chen, 2016, 2019; Guseman et al., 2017; Taniguchi et al., 2017; Ashraf et al., 2019; Furutani et al., 2020; Waite et al., 2020). In addition, *DRO1* orthologs in Arabidopsis were placed within the larger *IGT* gene family, with the *LAZY* and *TILLER ANGLE CONTROL* genes (Yoshihara et al., 2013; Hollender and Dardick, 2015; Guseman et al., 2017; Taniguchi et al., 2017; Ge and Chen, 2019). Keeping in view, the present study was designed to analyze the role of *DRO1* orthologs in potato (*S. tuberosum*) exerting similar functions, we initially cloned *StDRO1* and conducted a series of functional analyses to study the function of *StDRO1* for the alternation of root architecture and improvement for the drought stress tolerance.

## Materials and Methods

### Plant Materials and Mutant Detection

Arabidopsis ecotype Columbia (Col.) was used for the present investigation. The Arabidopsis T-DNA insertion mutant (SALK_201221C, Col. background) *Atdro1* was obtained from the Arabidopsis Biological Resource Center (Ohio State University, United States). Heterozygous mutants of *AtDRO1* were first identified, and the homozygous mutants were obtained from self-crossed progenies of the heterozygous parent. The gene-specific primers of left genomic primer (LP) and right genomic primer (RP) were utilized for genotyping; moreover, LBa1 was used as border primer of T-DNA (Supplementary Table 1). The potato cultivars Atlantic and Unica for tissue culture seedlings were provided by the Key Laboratory of Crop Genetic Improvement and the Germplasm Innovation of Gansu Agricultural University whereas, virus-free potato mini-tubers of both drought-sensitive “Atlantic” and drought-tolerant “Unica” cultivars were provided by the Dingxi Academy of Agricultural Sciences, the Gansu province.

### Cloning, Vector Construction, and Subcellular Localization Analysis of *StDRO1*

Gene-cloning primers of *StDRO1* were designed according to the potato reference sequence available in the NCBI^2^ database (LOC102585440 and XM_006361272.2), gateway technology (Invitrogen, Thermo Fisher Scientific) was employed to clone *StDRO1* into the expression vector (Supplementary Table 1). The full-length coding sequence of *StDRO1* fragments was amplified and cloned into the pDONR/Zeo entry clone vector, and the sequence was verified. Later, *StDRO1* was cloned into the expression vector *pBIB-BASTA-35s-GWR-GFP* and the sequence was verified. StDRO1-green fluorescent protein (GFP) fusion protein and GFP control were transiently expressed in tobacco (*Nicotiana tabacum*) leaves mediated by *Agrobacterium* GV3101. Fluorescence signals were observed under a laser confocal microscope (Zeiss LSM800).

### Transgenic Plant Generation

The expression vector *pBIB-BASTA-35s-StDRO1-GFP* was transferred into the Col. and *Atdro1* mutant by the floral dip method (Clough and Bent, 1998). Polymerase chain reaction (PCR) was used to identify T_1_ transgene-positive lines, forward primer was designed according to the 35 s promoter and *StDRO1* gene-specific as reverse primer (Supplementary Table 1). T_1_-positive lines were harvested individually and further sown in isolated pots to develop T_2_ and T_3_ generations, genotyping was carried out for each generation. Stable homozygous transgenic lines were identified and studied for subsequent phenotypic observation and drought tolerance analysis.

### Phenotype Observation and Determination of Physiological and Biochemical Indexes of Transgenic Plants

#### Root Phenotype Observation

Arabidopsis seeds were surface-sterilized with 75% (v/v) ethanol for 40 s, followed by 1% (v/v) NaClO for an additional 8 min and then washed with desterilized water six times. The washed seeds were placed on one-half-strength MS plates containing 0.8% (w/v) agar and 1% (w/v) sucrose. Seeds were vernalized at 4°C for 3 days and transferred to a growth chamber under a long-day condition (16 h of light and 8 h of dark) at 22°C. After 2 weeks of growth, the images of the root system were taken. All measurements were carried out using ImageJ.^3^

#### Determination of Physiological and Biochemical Indexes Under Drought Stress

Arabidopsis seeds were surface-sterilized with the similar procedure as described above and placed at one-half-strength MS plates with 0.8% (w/v) agar, 1% (w/v) sucrose, and 75 mM mannitol to simulate drought stress conditions (Murashige and Skoog, 1962). After 2 weeks of growth, physiological and biochemical indexes were determined. The activities of superoxide dismutase (SOD), peroxidase (POD), and catalase (CAT) were measured by the nitrogen blue tetrazolium photoreduction method, guaiacol colorimetric method, and ultraviolet absorption method (Beauchamp and Fridovich, 1971), respectively. In addition, the proline (Pro) content was determined by the acid ninhydrin color method (Irigoyen et al., 1992).

#### Phenotypic Observation of Aerial Parts

Nutrient soil and vermiculite were mixed at a 2:1 volume ratio and supplied to 10-cm diameter pots. Sterilized and vernalized Arabidopsis seeds were sown in pots containing nutrient soils, and the pots were placed in a greenhouse under a long-day condition (16 h of light and 8 h of dark) at 22°C. After 5 weeks of growth, aerial parts’ images were taken.

### RNA Extraction and Quantitative Real-Time Polymerase Chain Reaction of *StDRO1*

#### Potted Potato Growth Conditions

Nutrient soil and vermiculite were mixed at a 2:1 volume ratio and put into a 38-cm diameter pot. Virus-free mini-tubers were sown 5 cm below the soil surface. The pots were placed under field conditions at Gansu Agricultural University, normal agronomic practices were carried out throughout the growing period. After 65 days of growth, the whole plant, including roots, was carefully uprooted from the soil. Various tissues were quickly frozen in liquid nitrogen and stored at −80°C. The same procedure was repeated for all plants under investigation.

#### Potato Tissue Culture Growth and Treatment Condition

Stems (approximately 2 cm) of 1-month-old tissue culture plantlets were cut and transferred to sterilized glass jars containing MS medium. The jars were placed in a greenhouse under long-day conditions (16 h of light and 8 h of dark) at 22°C. After 4 weeks of growth, potato seedlings were collected and grown with 200 mM mannitol in liquid medium for 0, 2, 6, 12, and 24 h. After treatment, seedlings were quickly frozen in liquid nitrogen and stored at − 80°C.

#### RNA Extraction and Quantitative Real-Time Polymerase Chain Reaction

The RNA extraction kit (Tiangen) was used for the extraction of total RNAs from various potato tissues. About 5 μg of RNA was transcribed to cDNA using the ReverTra Ace^®^ qPCR RT Master Mix kit (TOYOBO). The resulting cDNAs, corresponding to 100 ng of total RNA, were then used as templates for quantitative real-time PCR by the StepOnePlus™ Real-Time PCR System (Applied Biosystems) utilizing the TB Green^®^ Premix Ex Taq II kit (Takara). The relative expression level was calculated utilizing the –ΔΔCt method, and ACTIN2 was used as an internal control. The primers used for real-time PCR are listed in Supplementary Table 1. The experimental procedures were the same as those reported previously by Sun et al. (2017).

#### Statistical Data Analysis

All experiments were repeated independently at least three times, and each sampling was analyzed separately. SPSS 20.0 software was used for statistical analyses, and statistically significant differences were measured by using Student’s *t*-test at * *p* < 0.05; ^**^ *p* < 0.01; ^***^ *p* < 0.001.

## Results

### Subcellular Localization and Tissue-Specific Expression Analysis of *StDRO1*

**FIGURE 1:**
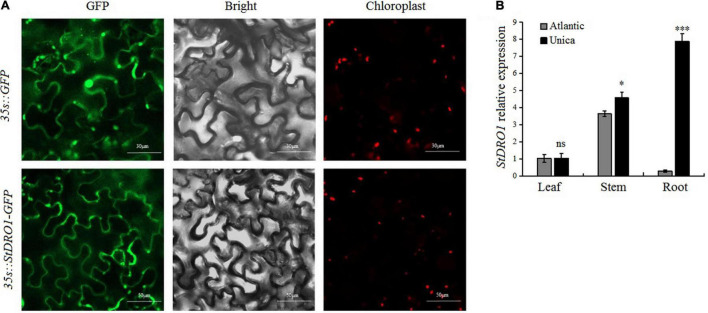
Subcellular localization and tissue-specific expression analysis of *StDRO1*. **(A)** Tobacco leaves transiently transformed with pBIB-BASTA-35s-StDRO1-GFP, the green fluorescent protein (GFP) fluorescence was mainly visible in the plasma membrane under a confocal laser scanning microscope. In tobacco leaves transformed with pBIB-35s-BASTA-GFP (control), GFP fluorescence was also visible in the cell nucleus. Left, GFP fluorescence; center, DIC images; right, chloroplast autofluorescence. **(B)** The expression level of *StDRO1* in the different tissues of potato cultivars. Sixty-five-day-old plant tissues of one drought-tolerant potato cultivar (Unica) and one drought-sensitive potato cultivar (Atlantic) were used. *StACTIN2* was used as a reference gene. Error bars represent SD (*n* = 3). Student’s *t*-tests were carried out to evaluate the significance of differences between the potato cultivar Atlantic and Unica for each examined organ. *p < 0.05; ^***^p < 0.001; and ns, not significant.

In the model plant Arabidopsis and some other plant species, *DRO1* was found to play an integral role for regulating the growth and development of the root system; however, its function in potatoes has not been reported (Uga et al., 2012; Guseman et al., 2017; Ashraf et al., 2019). The potato *StDRO1* was cloned, and an overexpression vector containing a StDRO1*-*GFP fusion was constructed. The vector was transiently transformed into tobacco leaves, and the GFP signals were observed under a laser confocal microscope. The StDRO1-GFP fusion protein was mainly located at the plasma membrane, whereas GFP control appeared in both the plasma membrane and the nucleus (Figure 1A). Moreover, the yeast two-hybrid assay displayed no interaction between potato *StDRO1* (or StDRO1ΔEAR) and StTOPLESS (Supplementary Figure 1).

We also evaluated *StDRO1* expression in the roots, stems, and leaves of the drought-tolerant potato cultivar (Unica) and the drought-sensitive potato cultivar (Atlantic). In both Atlantic and Unica, *StDRO1* expression was observed to be non-significant in leaves, whereas approximately 1.5-fold higher expression was observed in the stem of the Unica cultivar. However, the *StDRO1* expression level in the roots of Unica was highly significantly more than Atlantic (*p* < 0.001; *t*-test) (Figure 1B).

### *StDRO1* Regulates Plant Root Architecture

**FIGURE 2:**
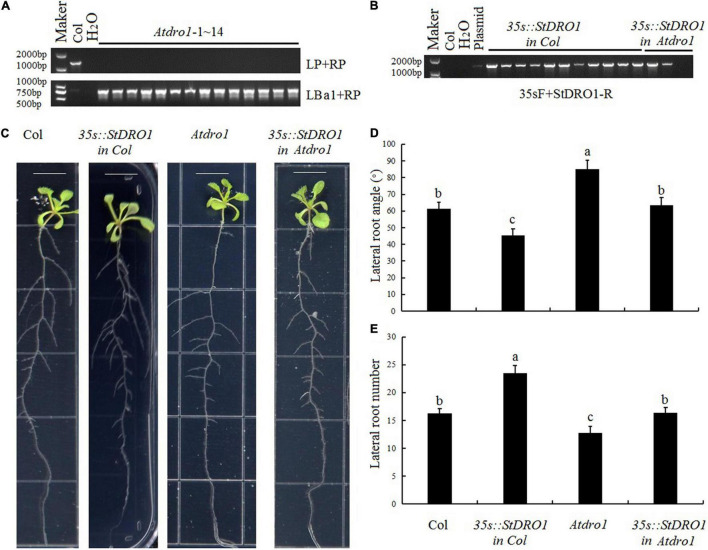
*StDRO1* regulates plant root architecture. **(A,B)** Genotyping of materials for four genotypes: Col. (negative control), *Atdro1* (here, 14 homozygous T-DNA insertion mutant individuals), *35s:StDRO1 in Col.* (11 positive transgenic lines for *StDRO1* expression in the Col. background), and *35s:StDRO1 in Atdro1* (2 positive transgenic lines for *StDRO1* expression in the *Atdro1* background). H_2_O was used as an additional negative control; the plasmid was used as a positive control; **(C–E)**: Images **(C)** and quantified lateral root angles **(D)** and numbers **(E)** of 2-week-old long-day-grown seedlings of Col., homozygous of *35s:StDRO1 in Col.*, *Atdro1*, and homozygous of *35s:StDRO1 in Atdro1*; the ruler length is 1 cm; Statistical tests (ANOVA) were carried out to evaluate the significance of differences among these four genotypes. Different letters indicate significant differences (*p* < 0.05).

To investigate the capacity of potato *StDRO1* to affect root architecture, we first obtained the Arabidopsis T-DNA insertion mutant *Atdro1* (Col. background) and confirmed the homozygous mutant (Figure 2A). Compared with Col. plants, *Atdro1* plants showed a significant increase in lateral root angle and a significant decrease in lateral root number, confirming that *AtDRO1* regulates root architecture. Moreover, we overexpressed *StDRO1* in both wild-type (Col.) and *Atdro1* mutant plants (Figure 2B). The results displayed that overexpressed *StDRO1* homozygous plants had significant reductions in lateral root angle and a significant increase in the number of lateral roots compared to Col [*p* < 0.05; analysis of variance (ANOVA)] (Figures 2C–E). In addition, we found complementary *Atdro1* mutant plants based on transgenic overexpression of *StDRO1* rescued root phenotypes as wild-type [*p* < 0.05; analysis of variance (ANOVA)] (Figures 2C–E). Thus, beyond confirming that Arabidopsis *AtDRO1* regulates root architecture, the results also indicated that potato *StDRO1* can regulate the angle and number of lateral roots.

**FIGURE 3:**
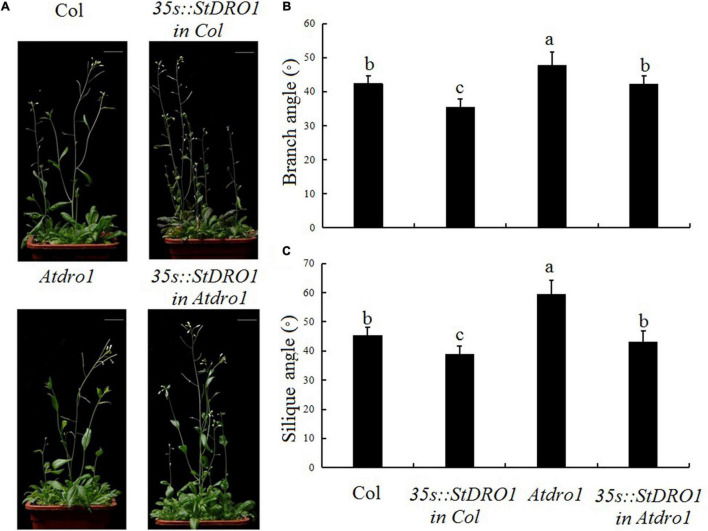
*StDRO1* regulates the branch angle of plant aerial organs. Images of **(A)** quantified branch angles **(B)** and silique angles **(C)** of 5-week-old plants of Col., homozygous of *35s:StDRO1 in Col.*, *Atdro1*, and homozygous of *35s:StDRO1 in Atdro1*. Statistical analysis (ANOVA) was carried out to evaluate the significance of differences between these four genotypes. Different letters indicate significant differences (*p* < 0.05).

Therefore, we observed the aerial organs of the different transgenic plants and found that the overexpression of *StDRO1* in the Col. background caused a significant decrease in the lateral branch angle and silique angle (*p* < 0.05; ANOVA) (Figure 3). In addition, *Atdro1* showed larger angles of side branches and siliques as compared to Col. Background, and genetic complementation analysis showed that the overexpression of *StDRO1* could successfully rescue the aerial organ phenotypes of *Atdro1* to the wild-type level (*p* < 0.05; ANOVA) (Figure 3).

### *StDRO1* Regulates the Drought Tolerance of Plants

**FIGURE 4:**
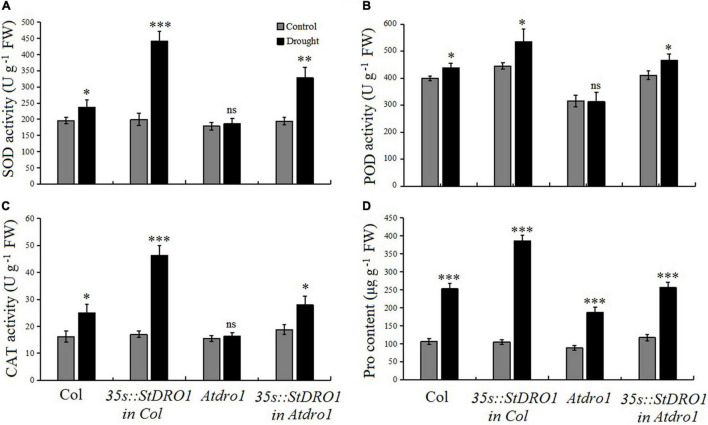
*StDRO1* regulates plant drought tolerance. **(A–D)** Responses of SOD, POD, and CAT activity along with proline (Pro) content under drought stress treatment. Two-week-old seedling of Col., homozygous *35s:StDRO1 in Col.*, *Atdro1*, and homozygous of *35s:StDRO1 in Atdro1* grown with or without 75 mM mannitol were used. Student’s *t*-test was carried out to evaluate the significant difference among control and drought treatment. *p < 0.05; ^**^p < 0.01; ^***^p < 0.001; and ns, not significant.

It has been reported that *OsDRO1* regulates the root architecture of rice and also influence rice to develop drought tolerance (Uga et al., 2013). Therefore, to examine the role of potato *StDRO1* in drought tolerance, we measured physiological and biochemical indicators of stress tolerance, including the activities of the antioxidant enzymes SOD, POD, and CAT, along with the Pro content of the four aforementioned Arabidopsis genotypes, under both normal growth conditions and drought treatments (75 mM mannitol in the growth medium). For Col. plants, drought stress increased the activities of the examined antioxidant enzymes and increased the Pro content (*p* < 0.05; *t*-test) (Figure 4). It was noteworthy that drought stress caused an increase in four indicators, namely, SOD, POD, CAT, and Pro content, and a highly significant increase in these indicators was observed in *StDRO1* overexpression line (35s:StDRO1 in Col.) plants than in Col. (*p* < 0.05; *t*-test). Moreover, our analysis of *Atdro1* plants showed no differences for SOD, POD, and CAT activities under normal and drought stress conditions; however, it detected a slight increase in Pro content in drought-stressed plants (*p* < 0.05; *t*-test). In contrast, complementation of *Atdro1* mutant plants based on transgenic *StDRO1* overexpression rescued the response for SOD, POD, CAT, and Pro content (*p* < 0.05; *t*-test) (Figure 4). These finding established the loss of *AtDRO1* function influences drought stress responses in Arabidopsis. The data also indicated that potato *StDRO1* can regulate drought tolerance in plants.

### *StDRO1* Gene Expression Is Induced by Drought Stress

**FIGURE 5:**
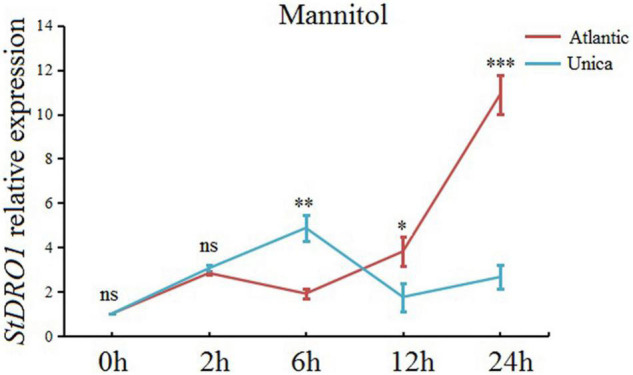
*StDRO1* gene expression is induced by drought stress. At the indicated time point, the effect of drought stress (by mannitol treatment) on *StDRO1* gene expression. Four-week-old seedlings of the drought-tolerant potato cultivar (Unica) and the drought-sensitive potato cultivar (Atlantic) grown under long-day-condition treated with or without 200 mM mannitol were used. *StACTIN2* was used as a reference gene. Error bars represent SD (*n* = 3). Student’s *t*-test was carried out to evaluate significance at each time point between Unica and Atlantic. *, *p* < 0.05; ^**^, *p* < 0.01; ^***^, *p* < 0.001; and ns, not significant.

Based on the recorded observation, *StDRO1* can regulate drought tolerance, we further expanded our research objective to investigate *StDRO1* gene expression under induced drought stress conditions. The real-time quantitative PCR (qRT-PCR) analysis results showed that drought treatment (mannitol), coupled with increasing treatment time, caused a slow elevation in the expression level of *StDRO1* for drought-sensitive Atlantic cultivar plants; however, it gradually increased by 10-fold with an additional 24-h sampling time point (Figure 5). In contrast, the drought-tolerant cultivar Unica showed that the expression level of *StDRO1* first increased and decreased subsequently, reaching its maximum at 6 h (with a 4-fold increase) (Figure 5). Thus, the *StDRO1* expression response to drought stress occurs earlier in the drought-tolerant cultivar Unica compared to the drought-sensitive cultivar Atlantic, and a significant difference of *StDRO1* expression at 6 h was detected between the two cultivars, (*p* < 0.01, *t*-test) (Figure 5).

## Discussion

Potato tubers are rich in starch, protein, vitamin C, crude fiber, potassium, calcium, and have an excellent nutritional profile (Zaheer and Akhtar, 2016; Robertson et al., 2018). Potatoes are grown worldwide and are of great significance for global nutrition and food security (Friedman, 2006). A series of studies have indicated that the spatial distribution of crop roots largely determines the ability of plants to obtain soil resources, regulating crop water, and nutrient use efficiency as well as crop adaptability to abiotic stress conditions (Malekpoor et al., 2014). In cereal crops, root traits have been extensively studied as an informative breeding index (Wasson et al., 2012; Lynch, 2013; Henry et al., 2014). In recent years, research on tuber crops has found that the optimization of root system architecture can confer substantial yield increases (Villordon et al., 2014b). However, drought is one of the principal abiotic stress limiting potato production around the globe (Deblonde and Ledent, 2001; Toubiana et al., 2020). For instance, the lack of water in the upper soil layers caused by irregular rainfall and high-intensity sunlight is common for rainfed potato planting areas. Therefore, maintaining tuber yield and commercial quality under such production conditions that have uneven distribution of water resources across different soil layers has been hotspot among researchers (Fabeiro et al., 2001; Liu et al., 2006; Kifle and Gebretsadikan, 2016; Li et al., 2019). In addition to studying and developing water-retaining and efficient cultivation techniques, researchers have sought to identify genes that help optimize root architecture and improve drought tolerance, which can be used in future potato molecular breeding programs.

In a study, map-based cloning was conducted on the shallow root rice variety “IR64” and the deep root variety “KP” for the *DRO1* gene, sequencing analysis revealed that a nucleotide deletion mutation occurred in the *DRO1* gene of “IR64” that caused premature cessation of DRO1 protein translation causing the deletion of C-terminal EAR motif (Uga et al., 2013; Guseman et al., 2017). For further verification, the near-isogenic line DRO1-NIL was constructed (having the “KP” *DRO1* allele in the “IR64” genetic background). Compared with “IR64,” DRO1-NIL has significantly smaller root angles at different growth stages, and higher yield under drought conditions without affecting root dry weight. The outcome of the study supported the hypothesis that *OsDRO1* participates in regulating the root angle and drought tolerance of rice (Uga et al., 2013). Furthermore, another group examined Arabidopsis and reported that single-gene mutations of *AtDRO1* can enlarge lateral root angles, showing that *AtDRO1* overexpression causes smaller lateral root and lateral branch angles. Taken together, this could indicate that *AtDRO1* regulates root architecture in Arabidopsis. The C-terminal EAR motif of the AtDRO1 protein was proven to be an essential element controlling root architecture (Guseman et al., 2017).

The construction, screening, and identification of transgenic lines in potato may took considerably longer time compared to Arabidopsis, therefore the *Atdro1* (Col. background) mutant was used to assess the potato *StDRO1* gene function. We found that the expression of potato *StDRO1* reduced the angles of lateral roots, side branches, and siliques; however, *StDRO1* expression increased lateral root numbers. In addition, transgenic expression of *StDRO1* could successfully rescue the defective phenotype of *Atdro1* mutant plants. We also observed that, under drought stress, the ability of *Atdro1* mutants to activate antioxidant enzymes and osmotic stress protection decreased, indicating that *AtDRO1* functions in drought stress responses in Arabidopsis, a result that has not been reported in previous studies.

Our findings based on transient expression in tobacco leaves indicated that the DRO1 protein is mainly localized at the plasma membrane. This membrane localization for *StDRO1* was reported in *OsDRO1* (rice) and *TaDRO1* (wheat) (Figure 1A) (Uga et al., 2013; Ashraf et al., 2019). A recent study reported that for *Atdro1* null mutant plants complemented with a VENUS-tagged *AtDRO1* driven by the native *AtDRO1* promoter, the reporter protein was localized in the nuclei of root tip cells (Waite et al., 2020). Further, the deletion of the EAR motif of DRO1 was reported to alter the localization of this protein in rice protoplasts (cell membrane with the full-length protein; cell nucleus and cytoplasm with the ΔEAR mutant variant) (Uga et al., 2013; Weber et al., 2014). EAR motifs are present in numerous transcriptional co-repressor proteins in plants, some of which have been shown to function by recruiting TOPLESS, a repressor of auxin-regulated, root-promoting genes (Kell, 2011). Previous reports in wheat complemented the interaction of TaDRO1-like with TaTOPLESS through the EAR motif with *in vitro* experiments (Ashraf et al., 2019). However, in the present investigation, the yeast two-hybrid assay displayed no interaction between potato StDRO1 (or StDRO1ΔEAR) and StTOPLESS, indicating that *DRO1* can putatively exert distinct molecular functions in different plant species. The detailed function and molecular mechanism of *StDRO1* in potato need to be further analyzed.

It is noteworthy to mention that in rice, Arabidopsis, and wheat, *DRO1* is mainly expressed in the root tips and basal part of shoots; however, in the present investigation we observed that *StDRO1* expression was low in leaves whereas it was strong in the stem part of the plant. In potato roots, the *StDRO1* expression level of the drought-tolerant cultivar Unica was significantly higher than that of the drought-sensitive cultivar Atlantic. Moreover, we also observed that the gene expression of *StDRO1* could be induced by drought stress, the transcriptional response of *StDRO1* to drought stress occurred significantly earlier in Unica than in the Atlantic cultivar. The findings were consistent with previous studies (Schafleitner et al., 2007; Kashiwagi et al., 2015; Li et al., 2019). These results strongly imply that *StDRO1* exerts the function of drought tolerance in potato; however, related molecular mechanism(s) await further characterization. Previous studies on Arabidopsis indicated that other members of the *IGT* gene family (to which DRO1 belongs) are involved in the regulation of root and shoot branching angles (Yoshihara et al., 2013; Taniguchi et al., 2017; Yoshihara and Spalding, 2017; Ge and Chen, 2019). Thus, our findings showing that the function of potato *StDRO1* for the regulation of root architecture and drought stress tolerance further support that *StDRO1* can be considered as an attractive gene for molecular breeding efforts to obtain robust-rooting and drought-tolerant potato varieties.

## Conclusion

In this study, DRO1 function for the regulation of root architecture and drought tolerance was investigated. In addition, *StDRO1* expression was several-fold higher in the stem and root of the Unica (drought-tolerant) cultivar, whereas, overexpression rescued the aerial organ and root phenotypes of the Arabidopsis *Atdro1* null mutant. The ectopic expression of *StDRO1* in Arabidopsis revealed a significant increase in biochemical indicators (e.g., SOD, POD, and CAT), along with Pro content under drought stress conditions, indicating that *StDRO1* is potentially a key player for potato drought stress tolerance. These results provide additional evidence that *StDRO1* functions during drought stress, thus laying a foundation for future studies focusing on *DRO1* and related genes in the drought responses of other crops under drought stress conditions.

## Data Availability Statement

The original contributions presented in the study are included in the article/Supplementary Material, further inquiries can be directed to the corresponding author.

## Author Contributions

CS designed the experiments and wrote the original draft of this manuscript and revision. CS, WL, KY, DX, and TQ performed the experiments and analyzed the data. SF, PK, ZB, YL, ZL, and JZ contributed to the review and editing. JB developed the research concept and managed the funding for the publication. All authors have read and agreed to the published version of this manuscript.

## Conflict of Interest

The authors declare that the research was conducted in the absence of any commercial or financial relationships that could be construed as a potential conflict of interest.

## Publisher’s Note

All claims expressed in this article are solely those of the authors and do not necessarily represent those of their affiliated organizations, or those of the publisher, the editors and the reviewers. Any product that may be evaluated in this article, or claim that may be made by its manufacturer, is not guaranteed or endorsed by the publisher.

## Footnotes

1
www.euroseeds.eu/potatoes

2
https://www.ncbi.nlm.nih.gov/

3
http://rsb.info.nih.gov/ij/

## References

[B1] Arai-Sanoh Y. Takai T. Yoshinaga S. Nakano H. Kojima M. Sakakibara H. (2014). Deep rooting conferred by DEEPER ROOTING 1 enhances rice yield in paddy fields. *Sci. Rep.* 4:5563. 10.1038/srep05563 24988911 PMC4080195

[B2] Ashraf A. Rehman O. U. Muzammil S. Léon J. Naz A. A. Rasool F. (2019). Evolution of Deeper Rooting 1-like homoeologs in wheat entails the C-terminus mutations as well as gain and loss of auxin response elements. *PLoS One* 14:e0214145. 10.1371/journal.pone.0214145 30947257 PMC6448822

[B3] Bartlett M. K. Sinclair G. Fontanesi G. Knipfer T. Walker M. A. McElrone A. J. (2022). Root pressure-volume curve traits capture rootstock drought tolerance. *Ann. Bot.* 129 389–402. 10.1093/aob/mcab132 34668965 PMC8944712

[B4] Beauchamp C. Fridovich I. (1971). Superoxide dismutase: improved assays and an assay applicable to acrylamide gels. *Anal. Biochem.* 44 276–287. 10.1016/0003-2697(71)90370-8 4943714

[B5] Brito G. G. Fagundes P. R. R. Telo G. M. Abreu A. G. Magalhães A. M. Jr. Franco D. F. (2016). Impact of Supra-Optimal Temperatures Onphysiology and Yield in Rice Field. *J. Agri. Sci.* 8:27. 10.5539/jas.v8n2p27

[B6] Cao M. Hengzhi L. Chao Z. Dongdong W. Xiaofang L. Qin C. (2020). Functional Analysis of StPHT1;7, a *Solanum tuberosum* L. Phosphate Transporter Gene, in Growth and Drought Tolerance. *Plants* 9:1384. 10.3390/plants9101384 33080882 PMC7650598

[B7] Chimungu J. G. Brown K. M. Lynch J. P. (2014). Large root cortical cell size improves drought tolerance in maize. *Plant Phys.* 166 2166–2178. 10.1104/pp.114.250449 25293960 PMC4256844

[B8] Clough S. J. Bent A. F. (1998). Floral dip: a simplified method for *Agrobacterium*-mediated transformation of Arabidopsis thaliana. *Plant J.* 16 735–743. 10.1046/j.1365-313x.1998.00343.x 10069079

[B9] Deblonde P. Ledent J. F. (2001). Effects of moderate drought conditions on green leaf number, stem height, leaf length and tuber yield of potato cultivars. *Eur. J. Agron.* 14 31–41. 10.1016/s1161-0301(00)00081-2

[B10] Edmeades G. O. (2013). *) Progress in Achieving and Delivering Drought Tolerance in Maize-An Update.* Ithaca, NY: ISAAA.

[B11] Fabeiro C. Olalla F. Juan J. (2001). Yield and size of deficit irrigated potatoes. *Agric. Water Manag.* 48 255–266. 10.3389/fpls.2017.01400 28848596 PMC5550687

[B12] Friedman M. (2006). Potato glycoalkaloids and metabolites: roles in the plant and in the diet. *J. Agric. Food Chem.* 54 8655–8681. 10.1021/jf061471t 17090106

[B13] Furutani M. Hirano Y. Nishimura T. Nakamura M. Taniguchi M. Suzuki K. (2020). Polar recruitment of RLD by LAZY1-like protein during gravity signaling in root branch angle control. *Nat. Commun.* 11:76. 10.1038/s41467-019-13729-7 31900388 PMC6941992

[B14] Ge L. Chen R. (2016). Negative gravitropism in plant roots. *Nat. Plants* 2:16155. 10.1038/nplants.2016.155 27748769

[B15] Ge L. Chen R. (2019). Negative gravitropic response of roots directs auxin flow to control root gravitropism. *Plant Cell Environ.* 42 2372–2383. 10.1111/pce.13559 30968964

[B16] Guseman J. M. Webb K. Srinivasan C. Dardick C. (2017). DRO1 influences root system architecture in Arabidopsis and Prunus species. *Plant J.* 89 1093–1105. 10.1111/tpj.13470 28029738

[B17] Henry A. Dixit S. Mandal N. P. Anantha M. S. Torres R. Kumar A. (2014). Grain yield and physiological traits of rice lines with the drought yield QTL qDTY(12.1) showed different responses to drought and soil characteristics in upland environments. *Funct. Plant Biol.* 41 1066–1077. 10.1071/fp13324 32481058

[B18] Hollender C. A. Dardick C. (2015). Molecular basis of angiosperm tree architecture. *New Phytol.* 206 541–556. 10.1111/nph.13204 25483362

[B19] Hoopes G. Meng X. Hamilton J. P. Achakkagari S. R. de Alves Freitas Guesdes F. (2022). Phased, chromosome-scale genome assemblies of tetraploid potato reveal a complex genome, transcriptome, and predicted proteome landscape underpinning genetic diversity. *Mol. Plant* 15 520–536. 10.1016/j.molp.2022.01.003 35026436

[B20] Irigoyen J. J. Emerich D. W. Sanchez-Diaz M. (1992). Water stress induced changes in concentrations of proline and total soluble sugars in nodulated alfalfa (*Medicago sativa*) plants. *Physiol. Plant* 84 55–60. 10.1034/j.1399-3054.1992.840109.x 11841302

[B21] Kashiwagi J. Krishnamurthy L. Purushothaman R. Upadhyaya H. D. Varshney R. K. (2015). Scope for improvement of yield under drought through the root traits in chickpea (*Cicer arietinum* L.). *Field Crops Res.* 170 47–54. 10.1016/j.fcr.2014.10.003

[B22] Kell D. B. (2011). Breeding crop plants with deep roots: their role in sustainable carbon, nutrient and water sequestration. *Annal. Bot.* 108 407–418. 10.1093/aob/mcr175 21813565 PMC3158691

[B23] Kifle M. Gebretsadikan T. G. (2016). Yield and water use efficiency of furrow irrigated potato under regulated deficit irrigation, Atsibi-Wemberta, North Ethiopia. *Agric. Water Manag.* 170 133–139. 10.1016/j.agwat.2016.01.003

[B24] Kondrák M. Marincs F. Antal F. Juhasz Z. Banfalvi Z. (2012). Effects of yeast trehalose-6-phosphate synthase 1 on gene expression and carbohydrate contents of potato leaves under drought stress conditions. *BMC Plant Biol.* 12:74. 10.1186/1471-2229-12-74 22646706 PMC3459809

[B25] Kumar S. Asrey A. Mandal G. (2007). Effect of differential irrigation regimes on potato (*Solanum tuberosum* L.) yield and post-harvest attributes. *Indian J. Agric. Sci.* 77 366–368.

[B26] Leisner C. P. Hamilton J. P. Crisovan E. Manrique-Carpintero N. C. Marand A. P. Newton L. (2018). Genome sequence of M6, a diploid inbred clone of the high-glycoalkaloid-producing tuber-bearing potato species Solanum chacoense, reveals residual heterozygosity. *Plant J.* 94 562–570. 10.1111/tpj.13857 29405524

[B27] Li X. Ramírez D. A. Qin J. Dormatey R. Bi Z. Sun C. (2019). Water restriction scenarios and their effects on traits in potato with different degrees of drought tolerance. *Sci. Hortic.* 256:108525. 10.1016/j.scienta.2019.05.052

[B28] Liao Q. Chebotarov D. Islam M. S. Quintana M. R. Natividad M. A. De Ocampo M. (2022). Aus rice root architecture variation contributing to grain yield under drought suggests a key role of nodal root diameter class. *Plant Cell Environ.* 45 854–870. 10.1111/pce.14272 35099814

[B29] Liu F. Ali S. Andersen M. N. Sven-Erik J. Jensen C. R. (2006). Physiological responses of potato (*Solanum tuberosum* L.) to partial root-zone drying: ABA signalling, leaf gas exchange, and water use efficiency. *J. Exp. Bot.* 57 3727–3735. 10.1093/jxb/erl131 16982651

[B30] Lynch J. P. (2013). Steep, cheap and deep: an ideotype to optimize water and N acquisition by maize root systems. *Annals Bot.* 112 34–357. 10.1093/aob/mcs293 23328767 PMC3698384

[B31] Lynch J. P. Brown K. M. (2012). Topsoil foraging-An architectural adaptation of plants to low phosphorus availability. *Plant Soil* 237 225–237. 10.1023/A:1013324727040

[B32] Malekpoor M. F. Seymour G. B. Swarup R. Moeiniyan B. H. Ramsey R. J. Thompson A. J. (2014). Environmental, developmental, and genetic factors controlling root system architecture. *Biotechnol. Genet. Eng. Rev.* 30 95–112. 10.1080/02648725.2014.995912 25652818

[B33] Manschadi A. M. Christopher J. Devoil P. Hammer G. L. (2006). The role of root architectural traits in adaptation of wheat to water-limited environments. *Fun. Plant Biol.* 33 823–837. 10.1071/FP06055 32689293

[B34] Mansoor-khani F. M. Seymour G. B. Swarup R. Bagheri H. M. Ramsey R. Thompson A. J. (2014). Environmental, developmental, and genetic factors controlling root system architecture. *Biotechnol. Gen. Eng. Rev.* 30 95–112. 25652818 10.1080/02648725.2014.995912

[B35] Murashige T. Skoog F. (1962). A revised medium for rapid growth and bio assays with tobacco tissue cultures. *Physiol. Plant* 15 473–497. 10.1111/j.1399-3054.1962.tb08052.x

[B36] Pieczynski M. Wyrzykowska A. Milanowska K. Boguszewska-Mankowska D. Zagdanska B. Karlowski W. (2018). Genome wide identification of genes involved in the potato response to drought indicates functional evolutionary conservation with Arabidopsis plants. *Plant Biotechnol. J.* 16 603–614. 10.1111/pbi.12800 28718511 PMC5787840

[B37] Porter G. A. Opena G. B. Bradbury W. B. McBurnie J. C. Sisson J. A. (1999). Soil management and supplemental irrigation effects on potato: I. Soil properties, tuber yield, and quality. *Agron. J.* 91 416–425. 10.2134/agronj1999.00021962009100030010x

[B38] Ranjan A. Sinha R. Singla-Pareek S. L. Pareek A. Singh A. K. (2022). Shaping the Root System Architecture in Plants for Adaptation to Drought Stress. *Physiol. Plant* 174:e13651. 10.1111/ppl.13651 35174506

[B39] Rasool F. Khan M. R. Schneider M. Uzair M. Aqeel M. Ajmal W. (2022). Transcriptome unveiled the gene expression patterns of root architecture in drought-tolerant and sensitive wheat genotypes. *Plant Physiol. Biochem.* 178 20–30. 10.1016/j.plaphy.2022.02.025 35247694

[B40] Robertson T. M. Alzaabi A. Z. Robertson M. D. Fielding B. A. (2018). Starchy Carbohydrates in a Healthy Diet: The Role of the Humble Potato. *Nutrients* 10:1764. 10.3390/nu10111764 30441846 PMC6267054

[B41] Schafleitner R. Gutierrez R. Espino R. Gaudin A. Pérez J. Martínez M. (2007). Field Screening for Variation of Drought Tolerance in *Solanum tuberosum* L. by Agronomical, Physiological and Genetic Analysis. *Potato Res.* 50 71–85. 10.1007/s11540-007-9030-9

[B42] Shahnazari A. Liu F. Andersen M. N. Jacobsen S. E. Jensen C. R. (2007). Effects of partial root-zone drying on yield, tuber size and water use efficiency in potato under field conditions. *Field Crops Res.* 100 117–124. 10.1016/j.fcr.2006.05.010

[B43] Smith S. De Smet I. (2012). Root system architecture: insights from Arabidopsis and cereal crops. *Philos. Trans. R. Soc. B. Biol. Sci.* 367 1441–1452. 10.1098/rstb.2011.0234 22527386 PMC3321685

[B44] Sun C. Ali K. Yan K. Fiaz S. Dormatey R. Bi Z. (2021). Exploration of Epigenetics for Improvement of Drought and Other Stress Resistance in Crops: A Review. *Plants* 10:1226. 10.3390/plants10061226 34208642 PMC8235456

[B45] Sun C. Yan K. Han J. T. Tao L. Lv M. H. Shi T. (2017). Scanning for New BRI1 Mutations via TILLING Analysis. *Plant Physiol.* 174 1881–1896. 10.1104/pp.17.00118 28461403 PMC5490892

[B46] Taniguchi M. Furutani M. Nishimura T. Nakamura M. Fushita T. Iijima K. (2017). The arabidopsis LAZY1 family plays a key role in gravity signaling within statocytes and in branch angle control of roots and shoots. *Plant Cell* 29 1984–1999. 10.1105/tpc.16.00575 28765510 PMC5590491

[B47] 3,000 rice genomes project (2014). The 3,000 rice genomes project. *Gigascience* 3:7. 10.1186/2047-217X-3-7 PMC4035669 24872877

[B48] Toubiana D. Cabrera R. Salas E. Maccera C. Franco Dos Santos G. Cevallos D. (2020). Morphological and metabolic profiling of a tropical-adapted potato association panel subjected to water recovery treatment reveals new insights into plant vigor. *Plant J.* 103 2193–2210. 10.1111/tpj.14892 32579242 PMC7540292

[B49] Uga Y. Hanzawa E. Nagai S. Sasaki K. Sato Y. T. (2012). Identification of qSOR1, a major rice QTL involved in soil-surface rooting in paddy fields. *Theor. Appl. Gen.* 124 175–186. 10.1007/s00122-011-1688-3 21894467

[B50] Uga Y. Sugimoto K. Ogawa S. Rane J. Ishitani M. Hara N. (2013). Control of root system architecture by DEEPER ROOTING 1 increases rice yield under drought conditions. *Nat. Genet.* 45:1097. 10.1038/ng.2725 23913002

[B51] Vicki L. C. Volker B. (2002). The Maize Genome Sequencing Project. *Plant Phys.* 130 1594–1597. 10.1104/pp.015594 12481042 PMC1540264

[B52] Villordon A. Q. Ginzberg I. Firon N. (2014a). Root architecture and root and tuber crop productivity. *Trends Plant Sci.* 19 419–425. 10.1016/j.tplants.2014.02.002 24630073

[B53] Villordon A. Q. Ginzberg I. Firon N. (2014b). Root architecture and root and tuber crop productivity. *Trend. Plant Sci.* 19 419–425. 10.1016/j.tplants.2014.02.002 24630073

[B54] Waite J. M. Collum T. D. Dardick C. (2020). AtDRO1 is nuclear localized in root tips under native conditions and impacts auxin localization. *Plant Mol. Biol.* 103 197–210. 10.1007/s11103-020-00984-2 32130643 PMC7170825

[B55] Walkowiak S. Gao L. Monat C. Haberer G. Kassa M. T. Brinton J. (2020). Multiple wheat genomes reveal global variation in modern breeding. *Nature* 588 277–283. 10.1038/s41586-020-2961-x 33239791 PMC7759465

[B56] Walworth J. L. Carling D. E. (2002). Tuber initiation and development in irrigated and non-irrigated potatoes. *Am. J. Potato Res.* 79 387–395. 10.1007/bf02871683

[B57] Wasson A. P. Richards R. A. Chatrath R. Misra S. C. Prasad S. V. Rebetzke G. J. (2012). Traits and selection strategies to improve root systems and water uptake in water-limited wheat crops. *J. Exp. Bot.* 63 3485–3498. 10.1093/jxb/ers111 22553286

[B58] Weber R. L. M. Wiebke-Strohm B. Bredemeier C. Margis-Pinheiro M. de Brito G. G. Rechenmacher C. (2014). Expression of an Osmotin-Like Protein from *Solanum nigrum* Confers Drought Tolerance in Transgenic Soybean. *BMC Plant Biol.* 14:343. 10.1186/s12870-014-0343-y 25492565 PMC4268879

[B59] Weigel D. Mott R. (2009). The 1001 Genomes Project for *Arabidopsis thaliana*. *Genome Biol.* 10:107. 10.1186/gb-2009-10-5-107 19519932 PMC2718507

[B60] Xie M. Chung C. Y. Li M. W. Wong F. L. Wang X. Liu A. (2019). A reference-grade wild soybean genome. *Nat. Commun.* 10:1216. 10.1038/s41467-019-09142-9 30872580 PMC6418295

[B61] Yoshihara T. Spalding E. P. (2017). LAZY Genes Mediate the Effects of Gravity on Auxin Gradients and Plant Architecture. *Plant Physiol.* 175 959–969. 10.1104/pp.17.00942 28821594 PMC5619908

[B62] Yoshihara T. Spalding E. P. Iino M. (2013). AtLAZY1 is a signaling component required for gravitropism of the Arabidopsis thaliana inflorescence. *Plant J.* 74 267–279. 10.1111/tpj.12118 23331961

[B63] Zaheer K. Akhtar M. H. (2016). Potato Production, Usage, and Nutrition–A Review. *Crit. Rev. Food Sci. Nutr.* 56 711–721. 10.1080/10408398.2012.724479 24925679

[B64] Zaki H. E. M. Radwan K. S. A. (2022). Response of potato (*Solanum tuberosum* L.) cultivars to drought stress under in vitro and field conditions. *Chem. Biol. Technol. Agric.* 9:1.

[B65] Zhao J. Zhan X. Jiang Y. Xu J. (2018). Variations in climatic suitability and planting regionalization for potato in northern China under climate change. *PLoS One* 13:e0203538. 10.1371/journal.pone.0203538 30260968 PMC6159864

