# Supplementary material for: Expression of Potato StDRO1 in Arabidopsis Alters Root Architecture and Drought Tolerance
Source: Front Plant Sci. 2022 May 19;13:836063. doi: 10.3389/fpls.2022.836063 (PMC9161210; doi:10.3389/fpls.2022.836063)
Supplement: Supplementary file 1 [file Data_Sheet_1.docx]

**Table S1.** Primer sequences used in this study.

| **Name** | **Primer sequence (5′-3′)** | **Application** |
| --- | --- | --- |
| LP | GAACGGGAGAAAAACCTTCTG | Arabidopsis *Atdro1* mutant (T-DNA Insertion) genotyping |
| RP | TGAGTGCTACGTTGTGAGCTG |  |
| LBa1 | TGGTTCACGTAGTGGGCCATCG |  |
| Clone-StDRO1-F | GGGGACAAGTTTGTACAAAAAAGCAGGCTTCTTCAAAGATTGGCCAGATTCCT | *StDRO1* cloning (attB sites are underlined) |
| Clone-StDRO1-R | GGGGACCACTTTGTACAAGAAAGCTGGGTCGGGCATTTTGGGGATGTATTTT |  |
| 35s-F | GAAACCTCCTCGGATTCCATTG | Identification of transgenic lines |
| StDRO1-R | AGGGGCATTTTGGGGATGTATT |  |
| qPCR-StDRO1-F | AATCAAGAATGGAGAAGCTTTTGAG | qRT-PCR analysis |
| qPCR-StDRO1-R | CACCCATTTAGATCCATCATAACAA |  |
| StACTIN-F | AGGAGCATCCTGTCCTCCTAA | qRT-PCR reference gene of potato |
| StACTIN-R | CACCATCACCAGAGTCCAACA |  |


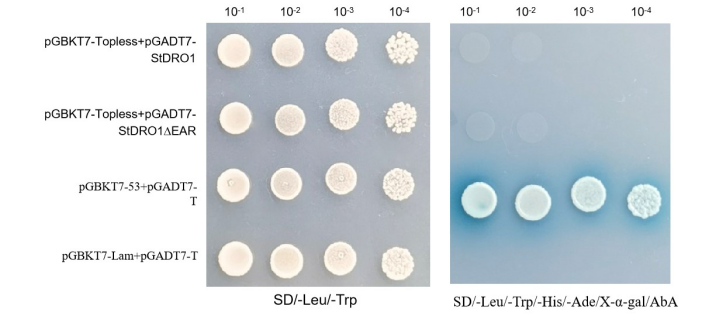


**Figure S1:** No interaction was detected between potato *StDRO1* (or StDRO1∆EAR) and StTOPLESS in a yeast two-hybrid assay using *StDRO1* (or StDRO1∆EAR) as the bait and StTOPLESS as the prey.
